# Vitamin E Circular Dichroism Studies: Insights into Conformational Changes Induced by the Solvent’s Polarity

**DOI:** 10.3390/membranes6040056

**Published:** 2016-12-14

**Authors:** Drew Marquardt, Brad J. Van Oosten, Mikel Ghelfi, Jeffrey Atkinson, Thad A. Harroun

**Affiliations:** 1 Department of Physics, Brock University, St. Catharines, ON L2S 3A1, Canada; brad.van.oosten2@brocku.ca; 2 Institute of Molecular Biosciences, Biophysics Division, NAWI Graz, University of Graz, Graz 8010, Austria; 3 BioTechMed-Graz, Graz 8010, Austria; 4 Department of Chemistry, Brock University, St. Catharines, ON L2S 3A1, Canada; mikel.ghelfi2@brocku.ca (M.G.); jatkinson@brocku.ca (J.A.)

**Keywords:** Vitamin E, *α*-tocopherol, *δ*-tocopherol, methylated tocopherol, circular dichroism, reichardt’s dye

## Abstract

We used circular dichroism (CD) to study differences in CD spectra between *α*-, *δ*-, and methylated-*α*-tocopherol in solvents with different polarities. CD spectra of the different tocopherol structures differ from each other in intensity and peak locations, which can be attributed to chromanol substitution and the ability to form hydrogen bonds. In addition, each structure was examined in different polarity solvents using the Reichardt index—a measure of the solvent’s ionizing ability, and a direct measurement of solvent–solute interactions. Differences across solvents indicate that hydrogen bonding is a key contributor to CD spectra at 200 nm. These results are a first step in examining the hydrogen bonding abilities of vitamin E in a lipid bilayer.

## 1. Introduction

**Figure 1:**
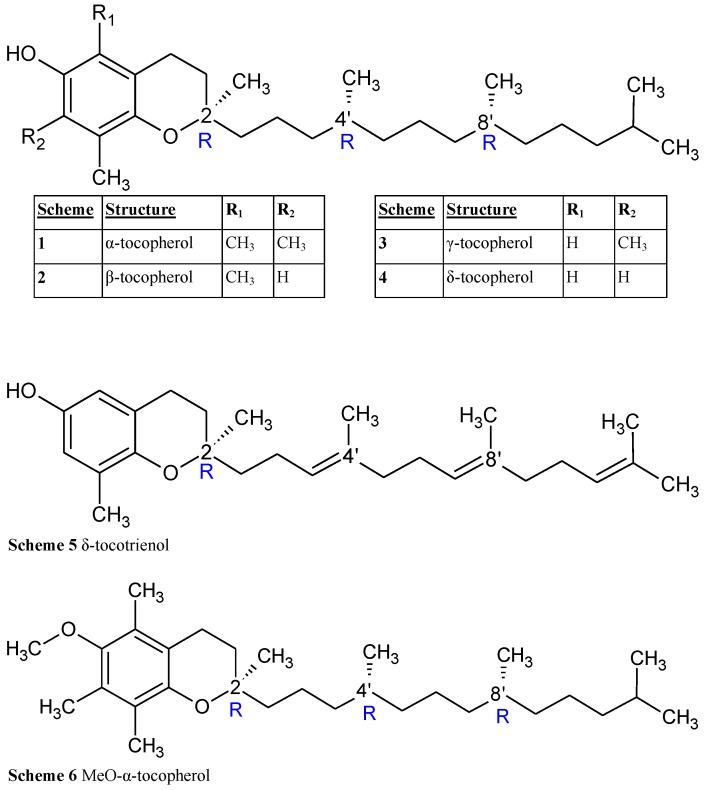
The chemical structures of the examined vitamin E compounds.

Viamin E is a liposoluble antioxidant, commonly understood to protect cell membranes from peroxidation reactions. Although rare, Vitamin E deficiency has been shown to lead to infertility [1] and neuromuscular dysfunction [2]. In industry, tocopherol acetate is extensively used as a preservative in cosmetics and foods. Vitamin E consists of three families comprised of as many as 12 compounds, differing primarily in their chromanol ring structure (*α*, *β*, *γ*, *δ* homologues, Figure 1 Schemes 1–4) and side-chain saturation (e.g., 5–6). Tocopherols have a fully saturated side-chain, tocotrienols have a isoprenoid derived side chain with three double bonds [3], and tocomonoenols contain a single site of unsaturation either at the isoprenoid chain terminus or along the isoprenoid chain itself [4]. The most common chromanol ring homologues differ in the methylation at two points on the chromanol ring, although single and non-methylated chromanols have also been found in nature [5]. Although all 12 members of Vitamin E have commonalities, and are all found in our diets, only *α*-tocopherol is taken up by the human anatomy for use.

Despite our extensive understanding of tocopherol’s oxidation chemistry, *α*-tocopherol’s (aToc) actual molecular role in the human body is still being debated. For example, Traber and Atkinson recently wrote that “all of the observations concerning the in vivo mechanism of action result from its role as a potent lipid-soluble antioxidant” [6], while Azzi makes the counter argument that “*α*-tocopherol is not able, at physiological concentrations, to protect against oxidant-induced damage” [7]. We believe that this ongoing debate regarding vitamin E’s biological role is the result of conflicting—and sometimes misinterpreted—data.

**Figure 2:**
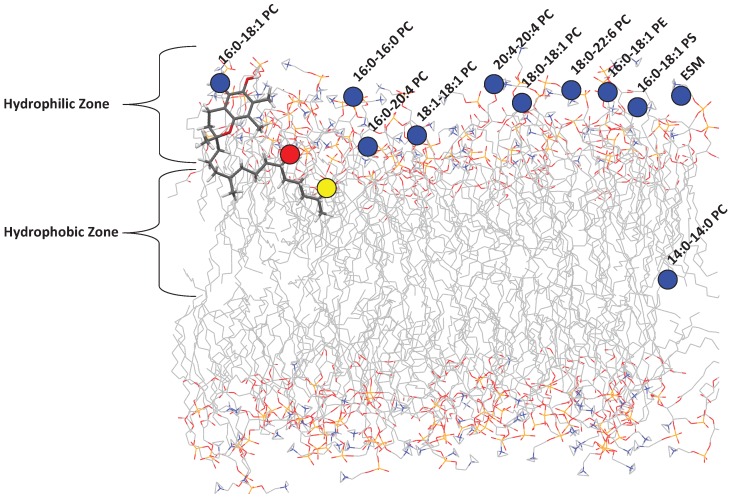
A representation of the locations that *α*-tocopherol (aToc) assumes in different phospholipid bilayers. The blue circles show the location of the aToc-C5d3 label in the vicinity of the lipid headgroups. The red circle is the location of the aToc-C5’d2 label, and the yellow is the aToc-C9’d2 label. Reproduced from [15].

It is well known that tocopherol incorporates itself into lipid membranes. Recently, Marquardt et al. reported on the location of aToc in a variety of phospholipid bilayers of differing composition (Figure 2) and its potential antioxidant capabilities using neutron diffraction, NMR spectroscopy, and UV/vis absorbance [8,9,10,11]. However, due to the disordered nature of phospholipid bilayers [12], the large biological diversity of phospholipids, and the high phospholipid concentration required, the circular dichroism (CD) of tocopherol inside bilayers was not investigated.

**Figure 3:**
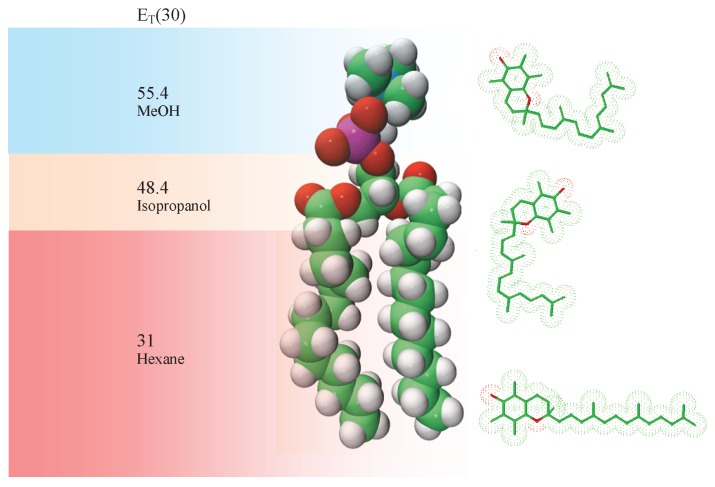
Cartoon representation of aToc’s location in a phospholipid bilayer along with the resulting polarity of the location and the representative solvent. Polarities along the lipid’s long axis were determined by Cohen, Afri, and Frimer [14].

In order to address this problem, we selected a number of common organic solvents (methanol ($E_{T}\left(30\right)$ = 55.4), isopropanol ($E_{T}\left(30\right)$ = 48.4), and hexane ($E_{T}\left(30\right)$ = 31)) which mimic the bilayer’s environment in the direction along the bilayer normal (i.e., perpendicular to the plane of the bilayer). We can then relate the observed specta to locations in the membrane in which aToc could reside, as shown in Figure 3. The polarity index utilized is an established method for measuring the polarity of solvents, and is based on the absorbance of Reichardt’s dye [13]. Previous work has been done on the assignment of $E_{T}\left(30\right)$ values to different parts of a phosphatidylcholine (PC) phospholipid bilayer [14].

CD is a powerful technique that probes the asymmetric configuration of electronic transitions of optically active molecules. Tocopherols have three chiral centers at carbons C-2, C-4’, and C-8’, and tocotrienols have one chiral center at carbon C-2. The naturally occurring stereoisomer has a configuration *2R,4’R,8’R*, originally named (+)-*α*-tocopherol. The epimer at the C-2 position can be obtained by a racemic or total synthesis, and was previously called (-)-*α*-tocopherol. Synthesis without stereochemical control produces *all-rac*-*α*-tocopherol. After purification, CD is used to determine the isomer of biomolecules obtained either through synthetic means or natural extraction [16].

A few CD spectra of tocotrienols, tocopherols, and tocopherol esters have been published over the years; however, to the best of our knowledge, there are no published electronic CD spectra of *α*-tocopherol, the most biologically active member of the vitamin E family [5,17]. In addition, there has been only one study in the mid-UV region (190–220 nm), where features of the CD signal increase significantly. Furthermore, there is a reported solvent dependence of the optical rotation of aToc that has not been explored since the original report by Baxter in 1943 [18].

Since tocopherol resides in cell membranes made up primarily of lipids and proteins, it may experience a range of polarities as it explores the membrane’s hydrophobic interior, including the polar water environment just outside of the membrane. We have recently determined that aToc is located at different positions in a model lipid bilayer, depending on the chemical composition of the bilayer. Three key locations were identified regarding aToc’s active oxygen, namely: (i) above the glycerol backbone and among the PC headgroups; (ii) close to the glycerol backbone; and (iii) at the centre of the phospholipid bilayer.

In this study, we make use of the chiral nature of RRR-*α*-tocopherol (aToc) and *δ*-tocopherol (dToc) in order to determine the conformational differences of these molecules in solvents of varying polarity, by means of synchrotron vacuum UV CD. The intensity of synchrotron radiation allows us to use solvents with a higher UV cutoff, and enables one to more accurately compare spectra in the far UV. The data show a modest solvent dependence associated with conformational changes of aToc and dToc, indicating that the conformation adopted by the C-2 chiral center depends on the polarity of the solvent. We also include the methylated analogue of aToc, effectively removing any influence that the hydroxyl group may have on the molecular configuration in the different polarity solvents.

## 2. Results and Discussion

**Figure 4:**
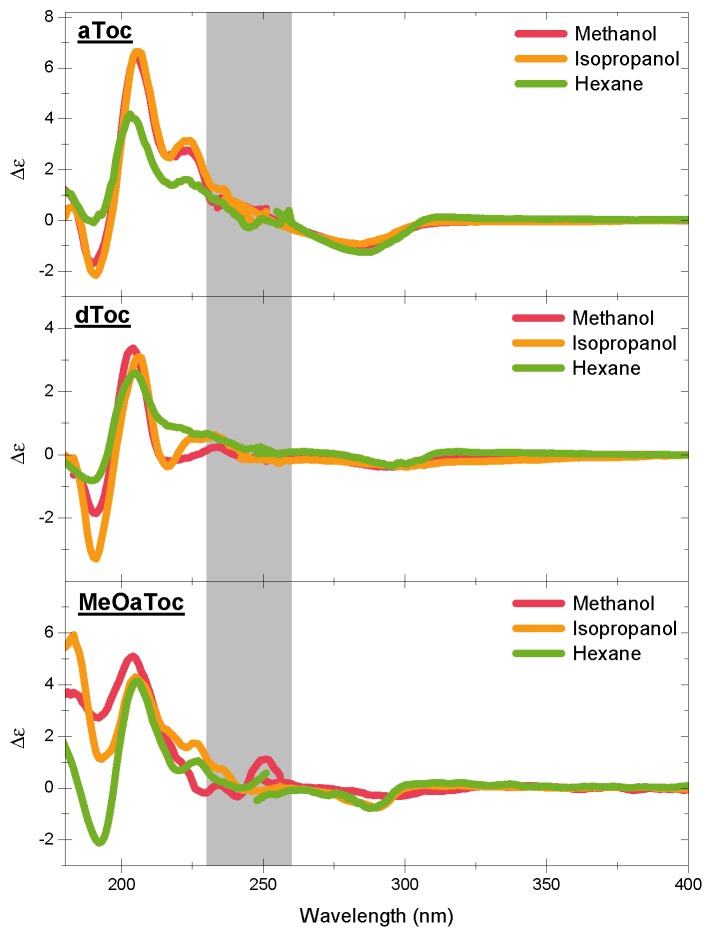
The measured CD spectra for *α*, *δ*, and methylated *α*-tocopherol (aToc, dToc, MeOaToc) in different solvents. The data are stitched together from two measurements (different wavelengths and pathlengths) by overlapping the common energy region between 230 and 260 nm (grey band). The line thickness corresponds to the error associated with the different measurements.

Figure 4 shows the high energy CD spectra of all conditions as a function of solvent. The features of the CD spectra are centered at the π−π UV absorption peaks of the aromatic chromophore, with all three compounds exhibiting similar characteristic features. The negative band near 300 nm is a well-known indicator of the overall stereoisomer of the molecule. Then, there are the alternating positive and negative bands near 210 and 190 nm, respectively.

**Figure 5:**
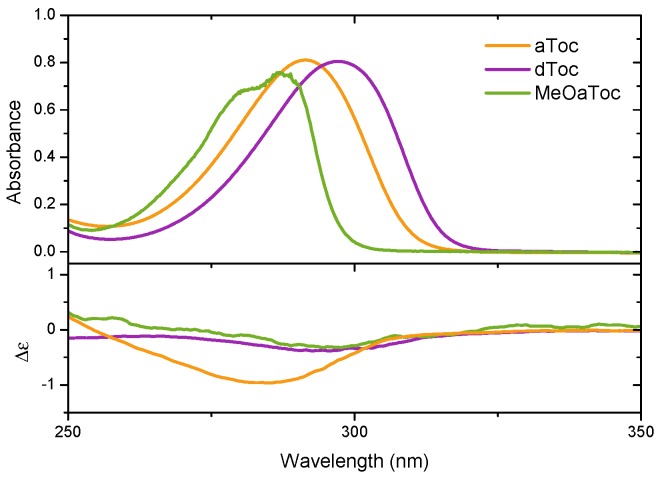
UV/vis spectrum (top panel) of aToc, dToc, and MeOaToc dissolved in methanol. The corresponding CD spectra are shown in the lower panel.

Figure 5 reports the medium UV absorbance and CD spectra of aToc, dToc, and MeOaToc in methanol. The data highlights the correspondence between the absorbance band and the measured molar ellipticity. Mazzini et al. reported the CD spectra of *δ*-tocopherol and tocotrienol homologues, as well as garcinoic acid (*δ*-tocotrienolic acid) in methanol in the near and medium UV range [17], and our spectra from dToc in methanol are in good qualitative agreement (discussed further below).

The spectral similarity of tocopherols and tocotrienols is to be expected; the chiral centre and chromophore should not differ significantly between the two molecules. However, the spectral intensity of tocopherol is considerably weaker than that of tocotrienol, which Mazzini attributes to exciton-coupling between the C-4’ alkene and C-2 arene centers of tocotrienol. Despite the inherently weaker signal of dToc, we were able to push further into the UV region and observe the negative CD return to 0 at ∼180 nm; this is a result of the significantly greater photon flux offered by synchrotron sources compared to the arc lamps of bench top instruments.

There are considerable similarities in overall shape and location between the different tocopherols (Figure 1, Schemes 1–4 and 6), but there are also several subtle differences that clearly demonstrate the effect that the solvent has on molecular conformation. For example, the aToc in methanol and isopropanol spectra are almost superimposable, with the methanol spectrum ever so slightly blue shifted. In contrast, in the case of aToc in hexane, the Δϵ intensity has decreased by a factor of $\frac{1}{2}$∼$\frac{1}{3}$, and its positive band experienced further blue shifting.

The differences in dToc spectra as a function of solvent are similar to aToc, but less pronounced. Although the positive ∼210 nm CD peaks are of similar intensity for methanol and isopropanol, the methanol sample is noticeably more blue-shifted (Δλ=1.5 nm). In addition, compared to isopropanol, the negative CD at 190 nm shows half the intensity in methanol. As with aToc, we see a decrease in CD signal in both peaks for the hexane, although the peaks are not as blue-shifted.

There is a positive shoulder in the aToc spectra in all three solvents in the 220–230 nm region. This shoulder is also present in dToc in hexane, and is likely the same band red-shifted, and is now centered at 230 nm in isopropanol. This band is greatly diminished to almost background levels in dToc in methanol—this is the first report of this band in the literature. Calculations by Mazzini suggest this to be the ${}^{1}$L${}_{a}$ chromane band; however, they claim it is below the larger ${}^{1}$B${}_{b}$ band. Although it does seem to be present in their dToc/methanol data, it does not play a significant role in the much larger tocotrienol signal, and therefore Mazzini et al. do not focus on it [17].

Comparing structures instead of solvents, we see that aToc’s CD signal at ∼200 nm is much stronger than dToc. The difference in intensities is consistent for both polar solvents; however, in hexane, aToc and dToc exhibit similar intensities. Furthermore, aToc remains blue-shifted across all solvents examined. When examining the negative CD signal at 190 nm, one observes the opposite trend than what is seen at 200 nm—i.e., increasing solvent polarity decreases the differences in CD signal between the different tocopherols.

For the O-alkylated MeOaToc, the polarity of the solvent had little effect on the intensity of the positive CD peak near 200 nm—although there is a slight peak shift to higher energy for the more polar solvents. This trend is also exhibited by aToc and dToc. The 220–230 nm shoulder is present for isopropanol, reduced in hexane, and becomes just slightly negative in methanol. The CD of MeOaToc does not differ significantly from the other tocopherols studied in the region of 240–300 nm.

By methylating the active oxygen, MeOaToc’s ability to hydrogen bond is negated. From the spectra of the hydrogen-bond-free MeOaToc, we see that the peak at 200 nm is largely unaffected in intensity by solvent polarity, suggesting that this peak is associated to hydrogen bonding by the active hydrogen. Likewise, removing the ability of the hydroxyl of either aToc or dToc to participate in hydrogen bonding (by solvating it in hexane) reduces the overall CD signal intensity. However, the greater loss of aToc intensity at the 200 nm peak, compared to dToc, may be a reflection of aToc’s higher oxidative reactivity [6]. Other spectroscopic techniques have observed aToc’s greater H-bonding capabilities; for example, Parker and Bisby observed both aToc radical sensitivity to the H-bonding and solvent polarity in the C=C stretching of the chromanol ring [19].

For MeOaToc in polar solvents, we presume that the solvation shells around its chromanol ring are disrupted, leading to observed differences in the region of 190 nm, where the band becomes positive with increasing solvent polarity. Mazzini et al. attribute the negative, high energy band seen in aToc and dToc to a valence to Rydberg electron transition, where it becomes positive with a switch from P-helicity (positive dihedral angle C-8a, O, C-2, C-3) to M-helicity of the chromane [17]. This raises the question of why the other bands do not change sign. Indeed, recent work by Batista et al. indicates that the overall signs of the B and L bands are primarily governed by the average conformation of the C-2 chiral-center, and whether or not the phytyl tail extends along the equatorial or axial directions, *not* the chromane half-chair helicity [20]. Thus, it is possible that the molecule experiences a change in helicity in response to solvent structure, without changing its chirality.

It is evident that the polarity of aToc’s environment has a significant effect on the CD signal when polarity differences are significant. Figure 4 illustrates that there is little difference between the CD spectra of aToc in methanol and in isopropanol. Previous Raman spectroscopy data indicate that the carbon–carbon stretching in the chromanol ring is similar in the two solvents, with the transition in hexane having a much lower energy carbon–carbon stretching [19]. In addition, it would seem that a non-polar environment mutes the differences in CD spectra among the different tocopherol structures. In the presence of a non-polar solvent, the spectra of the three structures appear to be practically the same, suggesting that the ability to H-bond is a contributing factor to the CD signal—MeOaToc has no hydrogen to donate. A decrease in polarity also causes a decrease and eventual disappearance of the shoulder at 230 nm. This shows that aToc assumes different physical properties across the bilayer, suggesting that its location could be directly related to its function.

Past studies of aToc in benzene (cyclic and aromatic) have reported that the specific rotations of aToc associated with the 546 nm region actually flips sign (positive to negative) [18]. In an effort to observe a flip in ellipticity to complement the rotation flip observed by Baxter et al., CD spectra of aToc in benzene and cyclohexane were collected. Despite our best efforts, we were not able to observe an aToc ellipticity change in benzene. In fact, there is no sign of ellipticity flipping for any of the studied tocopherols in benzene or cyclohexane (data not shown). Although interesting, this observation is not inconsistent with those of Baxter et al.

In an effort to make a connection between vitamin E’s locations and its in vivo activity, we have demonstrated that the molecule adopts different conformations in environments with different polarities. The next logical progression of this study is to examine the conformations of tocopherols in phospholipid dispersions. However, there are several technical hurdles that need to be overcome.

In conclusion, we have demonstrated that CD can be utilized to determine vital information regarding tocopherol’s conformation in different chemical environments. Due to CD’s rapid collection of data and sensitivity, the potential for studying chemical properties of aToc and other chiral antioxidants is intriguing (see Table 1).

## 3. Methods

### 3.1. Experimental Secton

All solvents were of reagent grade, and were used without any further purification. (+)-*α*-tocopherol (aToc) was purchased from Cole-Parmer (Vernon Hills, IL, USA). *δ*-tocopherol (dToc) was purchased from Sigma, and was purified on silica, eluting with 9:1 hexane:ethyl acetate. The (R)-6-methoxy-2,5,7,8-tetramethyl-2-((4R,8R)-4,8,12-trimethyltridecyl)chroman (MeOaToc) was prepared by adding sodium hydride (60% in mineral oil; 42 mg, 0.95 mmol) at room temperature in three aliquots to a solution of alpha-tocopherol (R,R,R) (450 mg, 0.95 mmol) in dry tetrahydrofuran (THF) (3 mL) under nitrogen atmosphere. After 10 min, methyl iodide (148.5, 0.95 mmol) was added at 40 ${}^{\circ }$C, and stirred for 2 h under nitrogen atmosphere. The reaction was quenched with water and extracted three times with dichloromethane. The organic phases were combined and dried over anhydrous sodium sulfate. The crude product was purified by chromatography on silica gel, using an elution gradient of hexane/ethyl acetate 6:1 to 1:1, which afforded 250 mg (54%) product. Based on mass spectroscopy, ${}^{1}$H NMR and thin layer chromatography (TLC) plating, the purtiy is estimated >95%.

In order to measure the CD spectra at higher photon energies, CD data were collected at the National Synchrotron Light Source U11 beam-line located at the Brookhaven National Laboratory (Upton, New York, NY, USA). Samples were made to a concentration ∼1 mg/mL, which allowed for the optimal absorbance of 1 at 200 nm. The samples were then injected into a custom-made cylindrical cuvette of pathlength 40 μm (Brock University, St. Catharines, ON, Canada). The windows of the cuvette were constructed of ${\mathrm{CaF}}_{2}$, allowing for data to be collected in the deep UV. Scans used a time constant 5 s and bandwidth of 1 nm from a Wadsworth monochromator.

CD spectra were measured on a Jasco J-600 CD spectrometer using 1 mm quartz cuvette (*λ* = 500 nm–220 nm) with a sensitivity of 10 mdeg, a bandwidth of 2 nm, and a scan rate of 50 nm/min. Tocopherol concentrations varied from 1–2 mg/mL for the optimal absorbance. UV/vis absorbance measurements were performed on a Ocean Optics USB4000 using a ISIS light source. Concentrations of tocopherol samples were confirmed by the UV/Vis absorbance at 290 nm.

## Figures and Tables

**Table 1:** Molar extinction coefficients (M${}^{-1}$ cm${}^{-1}$) at 290 nm.

Tocopherol	Methanol	Isopropanol	Hexane
aToc	2788 ± 36	2891 ± 36	3340 ± 50
dToc	2782 ± 42	3263 ± 20	3018 ± 42
MeOaToc	2885 ± 38	2059 ± 20	2375 ± 54
